# Patient perspectives on how to optimise benefits from a breathlessness service for people with COPD

**DOI:** 10.1038/s41533-020-0172-4

**Published:** 2020-04-08

**Authors:** Tim Luckett, Mary M. Roberts, Tracy Smith, Vinita Swami, Jin-Gun Cho, John R. Wheatley

**Affiliations:** 1 University of Technology Sydney (UTS), Faculty of Health, Improving Palliative, Aged and Chronic Care through Clinical Research and Translation (IMPACCT), Sydney, NSW Australia; 2 Department of Respiratory and Sleep Medicine, Westmead Hospital, Westmead, NSW Australia; 3 Ludwig Engel Centre for Respiratory Research, Westmead Institute for Medical Research, Westmead, NSW Australia; 4 The University of Sydney at Westmead Hospital, Westmead, NSW Australia

**Keywords:** Respiratory signs and symptoms, Chronic obstructive pulmonary disease

## Abstract

This study aimed to inform understanding of how to optimise patient-perceived benefits from a breathlessness service designed for patients with moderate to very severe chronic obstructive pulmonary disease (COPD). The Westmead Breathlessness Service (WBS) trains patients to self-manage over an 8-week programme, with multidisciplinary input and home visits. A qualitative approach was taken, using semi-structured telephone interviews. Each transcript was globally rated as suggesting ‘significant’, ‘some’ or ‘no’ impact from WBS, and thematic analysis used an integrative approach. Forty-one consecutive participants were interviewed to reach ‘information power’. Eighteen (44%) participants reported ‘significant’ impact, 17 (41%) ‘some’ impact, and two (5%) ‘no’ impact. Improvements to breathlessness were usually in the affective and impact dimensions but, more uncommonly, also sensory-perceptual. Participants who benefited in self-esteem, confidence and motivation attributed this to one-to-one multidisciplinary coaching and home visits. Further research should test whether including/excluding more intensive programme elements based on individual need might improve cost-effectiveness.

## Introduction

Chronic breathlessness is a common and debilitating symptom across many life-limiting conditions, including respiratory disease (e.g. chronic obstructive pulmonary disease (COPD)), heart disease and cancer, and often persists despite optimised, guideline concordant pharmacological treatment of the underlying medical condition(s)^1^. Chronic breathlessness includes at least three dimensions: ‘sensory-perceptual’ (severity and quality), ‘affective’ (unpleasantness and distress) and ‘impact’ (effects on everyday life)^2^. Over and above chronic breathlessness on a daily basis, many people experience episodes of acute-on-chronic breathlessness^3^. These episodes are associated with high levels of anxiety/panic and often precipitate Emergency Department (ED) presentations, many of which are not clinically indicated and represent poor utilisation of healthcare resources^4,5^.

While chronic breathlessness cannot be cured, it can be managed with non-pharmacological and pharmacological therapies aimed at modulating the perception of breathlessness and the individual’s response^6^. These therapies are ideally delivered within a self-management framework in which people learn skills to reduce the impact on functioning and sustain emotional wellbeing ^7,8^.

Randomised controlled trials (RCTs) from the UK have tested the effectiveness of five ‘breathlessness services’ in which expert multidisciplinary teams train patients to self-manage their chronic breathlessness using pharmacological and non-pharmacological therapies. These interventions have varied in duration and number/disciplines of healthcare professionals involved. Three services focused on people with lung cancer^9–11^, while another three included people with advanced cancer of any tumour type and/or non-malignant respiratory disease^12–14^. All except one trial have demonstrated efficacy for improving breathlessness-related outcomes^9–12^; Farquhar et al.^13,14^ found the Cambridge Breathlessness Intervention Service (CBIS) reduced distress due to breathlessness for patients with advanced cancer but not non-malignant respiratory disease^13,14^. However, a qualitative sub-study of the CBIS found that most patients with non-malignant respiratory disease perceived at least some benefit, including a reduction in anxiety/panic and increased confidence in managing their breathlessness^14^.

A review by Bausewein et al.^15^ compared and contrasted characteristics of the above five services together with their own untested Munich Breathlessness Service, and identified variability with regard to: intensity and duration (1–8 sessions over 1–12 weeks); location and mode (face-to-face in the clinic, at home and/or via telephone); individual versus group delivery; and the disciplines and specialties involved (especially the degree to which physicians were involved and a respiratory or palliative care perspective was emphasised)^15^. Thus far, there has been only one attempt to explore the influence of these variables on service efficacy. Johnson et al. found one session delivered by a single nurse or allied health professional was as effective as three sessions for reducing worst breathlessness over the past 24 h in patients with lung cancer, questioning whether more resource-intensive services are cost-effective^11^.

The current article reports on a new Australian breathlessness service developed specifically for people with moderate to very severe COPD (defined as FEV_1_/FVC ≤ 70%; FEV_1_ ≤ 60% predicted) and run over 8 weekly sessions (plus pre and post assessments) through a respiratory medicine outpatient service at a metropolitan hospital, which provides tertiary and quaternary services to a socio-economically and culturally diverse population. Of the breathlessness services reported to date^9–14^, the Westmead Breathlessness Service (WBS) is among the more resource intensive. The WBS programme is highly multidisciplinary by including a physician (0.2 full-time equivalent; FTE), two nurses (total 0.4 FTE), physiotherapist (0.3 FTE), occupational therapist (0.3 FTE) and dietitian (0.2 FTE), and offers in-home as well as outpatient individualised sessions. Supplementary Table 1 contains a week-by-week breakdown of WBS programme content. Referrers to WBS are asked to encourage patients to complete pulmonary rehabilitation (where feasible) prior to enrolment, presenting an opportunity to assess the degree to which patients perceive that the breathlessness service adds value to an established group intervention with high-level evidence for efficacy^16^.

Recruitment for a RCT evaluating WBS is currently underway, with the primary endpoint being mastery of breathlessness as measured by the Chronic Respiratory Questionnaire^17^. The current article reports on results from a qualitative sub-study of the trial, which aimed to develop an in-depth understanding of patient perceptions regarding the nature of any benefits derived from the service and components deemed most useful. To build on evidence to date, we were interested to replicate and extend findings from the qualitative study of CBIS^13,14^ by further exploring factors associated with perceived benefit, WBS’s perceived additional value compared to pulmonary rehabilitation, the degree to which patients perceived benefits to be associated with multidisciplinary care and home visits, and perceptions regarding the frequency and duration of sessions. We were also interested to learn from patients about ways in which they perceived the service might be improved, including the views of patients who derived limited benefit.

## Results

Information power was deemed to have been reached after interviews with 41 participants.

Two patients who completed the WBS programme were unintentionally omitted from the sample due to a miscommunication between clinical staff and the researcher conducting the interviews.

Participants had a mean age of 70.2 years (standard deviation 7.1) and were predominantly men (*n* = 24, 59%). See Table 1 for other participant characteristics and Supplementary Table 2 for illustrative quotes for each section below. Interviews ranged from 10.3 to 29.5 min.

**Table 1:** Characteristics of participants interviewed about their experiences of the Westmead Breathlessness Service (*n* = 41).

Characteristic (summary measure)	
Age (years)	70.2 (7.1)
Male (%)	59
Lives alone (%)	39
LTOT (%)	12
Completed PR within past year (%)	34
FEV_1_ (L)	0.79 (0.29)
FEV_1_ (% predicted)	31 (11)
VC (L)	2.27 (0.76)
VC (% predicted)	66 (15)
FEV_1_/VC (%)	36 (11)
6MWD (m)	296 (102)
mMRC (a.u.)	3.41 (0.87)
0–10 NRS on exertion (a.u.)	7.8 (1.8)

Data are expressed as mean (standard deviation) unless otherwise stated.

*LTOT* long term oxygen therapy, *PR* pulmonary rehabilitation, *FEV*_*1*_ forced expiratory volume in one second, *L* litres, *VC* vital capacity, *6MWD* 6 min walk distance, *m* metres, *mMRC* modified Medical Research Council Dyspnoea Scale, *NRS* numerical rating scale, *a.u.* arbitrary units.

### Patient perceptions of overall benefit from WBS

Overall, 37 of the 41 participant transcripts were rated for overall benefit, with results presented in Table 2. Four of the transcripts were considered difficult to code with confidence, either because they contradicted themselves regarding improvements (*n* = 3) or had provided insufficient information to rate (*n* = 1).

**Table 2:** Participant ratings of various levels of benefit after completing the 8-week Westmead Breathlessness Service programme (*n* = 41).

Level of benefit	Number (%)
Significant impact (Level 1)—clearly stated WBS made a difference	18 (44)
Some impact (Level 2)—no major change noted but valued specific aspects	17 (41)
No impact (Level 3)	2 (5)
Difficult to code	4 (10)

Most (*n* = 13/18) participants who were classified as deriving significant benefit (Level 1) cited improvements in activities of everyday living (ADLs) (e.g. housework, shopping), either in terms of being able to do new activities or perform tasks for a longer duration or with enhanced capability. For a third of participants with significant benefit, this flowed on from a perceived reduction in the severity of their breathlessness. For the remainder, benefit was characterised as a reduction in the impact and affective domains of breathlessness described in terms of greater exercise tolerance, faster recovery, less fear, and increased confidence and ability to cope.

Other reasons that participants were classified as deriving significant impact were related either to reports that they had successfully avoided unnecessary ED presentations or hospitalisations as a result of strategies they had learned through WBS, or had significant improvements in emotional wellbeing. The latter seemed to stem from a greater understanding and acceptance of COPD, as well as a sense of being cared for and feeling less alone.

Participants were usually classified as receiving some (Level 2) impact because they described benefit in terms of increased comfort in, rather than capacity for, ADLs.

The four participants rated as perceiving no (Level 3, *n* = 3) or inconsistent (difficult to code, *n* = 1) benefit, nonetheless praised the caring attitudes of WBS team members and struggled to identify ways that the service could be improved.

### Extension of themes from the CBIS evaluation

All benefits identified within the CBIS qualitative analysis^13,14^ were corroborated by at least some participants within the WBS sample, although there were a small number of negative cases for each (i.e. participants whose perspectives differed from the majority). While all the non-pharmacological therapies were beneficial for some participants, the proportion who reported benefit from each varied. A majority of participants who reported global benefit credited at least some role to learning about breathing techniques (*n* = 30/35) and the hand-held fan (*n* = 25/35). A smaller majority reported benefiting from learning about pacing (*n* = 19/35), and a minority from relaxation techniques (*n* = 10/35). Breathing techniques and the hand-held fan were perceived to provide psychological benefits as well as to positively influence breathlessness.

Exercise was perceived as yielding benefits indirectly rather than directly, by preventing deconditioning. The indirect nature of benefit and physical challenge associated with exercise made adherence difficult for many participants, with goal setting and motivation from the WBS team members often emphasised as important in enabling this.

As with the CBIS data, there was a substantial emphasis placed on how the service was delivered as well as its contents, especially the supportive ‘can do’ attitude of the WBS team.

### Patient perceptions of WBS versus pulmonary rehabilitation

Most (*n* = 26/34) participants who had previously undertaken pulmonary rehabilitation reported that the WBS programme was different. Compared with pulmonary rehabilitation, the WBS programme was perceived to cover a greater variety of therapies in more depth, be more personalised, involve caregivers more, and go beyond the physical aspects of COPD to more holistically address wellbeing and consider comorbidities such as anxiety.

Of four participants who perceived that WBS did not teach new skills over and above pulmonary rehabilitation, one was among those who perceived no benefit globally, and a second attributed WBS with helping to reinforce messages and motivate adherence to exercise.

### Patient perceptions regarding home visits

A key feature of the WBS programme that separated it from pulmonary rehabilitation for many participants was the availability of home visits. Around half (*n* = 20) of the participants perceived home visits to be an important feature of the service both for improving access and enabling tearm members to assess and remediate ADLs in situ. Two participants asserted that they could not have attended the service if it had been run exclusively at the hospital due to logistic problems with transport, limitations in mobility/physical functioning, or anxiety related to travel or hospitals. However, there were two exceptions, who reported feeling overwhelmed by the number and frequency of home visits.

### Patient perceptions of WBS’s individual focus

Participants often cited the individualised nature of the WBS programme as beneficial for enabling the WBS team to develop a deep understanding of their individual needs required to tailor advice, and to build personal relationships that communicated care and support and encouraged disclosure. Experience of being cared for and supported as an individual was among the most commonly emphasised feature of the service and perceived by many to be therapeutic in its own right. The characteristics of care and support that were most commonly accentuated included genuineness, lack of time constraint, shared understanding about the impacts of breathlessness, respect and dignity, and lack of judgement regarding smoking history. In some cases, this was contrasted with a lesser sense of support they had experienced in other healthcare encounters.

While two participants reported benefitting from socialising with other participants in previous group programmes such as pulmonary rehabilitation, all who were asked considered the benefits of one-to-one time to outweigh this.

### Patient perceptions regarding WBS’s programme duration

Everyone who was asked expressed approval regarding the duration of the programme, except for two participants who would have preferred a longer duration and three who perceived the content to be unnecessarily repetitive and/or include content not relevant to them. This last group differed from other participants who perceived repetition of content to be useful for consolidation and reinforcement. Many (*n* = 17) participants stressed the importance of knowing that the WBS team was available by telephone after the sessions were completed to give them a sense of continuing support and safety in the event of future problems.

### Patient perceptions of WBS multidisciplinary input

Participants varied in the degree to which they distinguished the roles and contributions of different disciplines within the WBS team. Some participants perceived that team members’ different skillsets worked in harmony together by focusing on different aspects of care and reinforcing key messages.

Even where participants seemed less clear on the role of each discipline, collective attention from a number of health professionals increased their sense of being cared for and motivated them to make more effort to self-manage. In this way, participants drew an explicit causal pathway from the team’s caring and supportive approach through to their capacity to self-manage and the resulting impacts on breathlessness.

Changes in participant self-esteem, confidence and motivation seemed not to be limited to breathlessness self-management but impacted their lives more generally.

While all the disciplines were singled out for praise, participants seemed especially impressed with the time commitment and level of care shown by the doctor.

Participants also highlighted the unique role the doctor played in prescribing medications (including low-dose opioids), ordering and interpreting tests, and using medical authority to enable access to healthcare for comorbid conditions and disability benefits.

Although not explicit, there was a sense that the doctor’s opinion carried special weight, especially on sensitive matters such as the need for referral to a psychologist or advance care planning.

There were also three negative cases who seemed not to perceive substantial differences in the roles of WBS team members. For the participant with no global benefit, this extended even to the doctor’s role.

### Patients’ suggestions for improving WBS

When asked how to improve WBS, more than half (*n* = 25) of participants responded by saying this would be difficult to do because quality was already so high. Everyone who volunteered improvements (*n* = 16) cited problems with travelling to/from the hospital due to a lack of means of transport or difficulty parking. While home visits were perceived to partially offset these problems, hospital visits were still required for initial assessment and one follow-up. At least one participant was unaware of the free patient transport service available to/from the hospital.

A small number of participants also recommended that WBS be made more widely available, either by including people with earlier stage disease (*n* = 1) or by better promoting it to those who were currently eligible but may not have heard of it (*n* = 2). Two participants suggested running WBS as an inpatient service during the time that patients were admitted for an exacerbation. Participants asking for the service to be extended in these ways usually qualified their suggestions by acknowledging that resources might be too limited to accommodate.

## Discussion

This study extends previous qualitative research on the nature of benefits that patients perceive from attending breathlessness services^13,14^, focusing for the first time on a service designed exclusively for patients with COPD. Patients perceived benefits across the affective, impact and (less commonly) sensory-perceptual dimensions of breathlessness, with some patients reporting benefits to extend to other facets of wellbeing more generally. Participants generally found it difficult to identify opportunities for improving the service, citing a high level of satisfaction. However, some participants perceived there to be opportunities to improve access to WBS by increasing awareness of the programme and alleviating transport/parking issues.

Participants generally perceived benefits to be associated with WBS’s multi-week duration, multidisciplinary input (including medical) and individualised administration via home visits, and also reported that the service added substantial value following pulmonary rehabilitation. Patients’ emphasis on the importance of home visits was especially noteworthy given that WBS is a metropolitan service, suggesting that, even with a relatively short distance to travel, many patients with COPD struggle to attend hospital appointments. Our research suggests that even some metropolitan patients might benefit from telehealth, which is currently funded by the Australian healthcare system only for regional and rural areas.

The proportion of participants reporting benefit from WBS was similar to those reported for the 2-week CBIS and 6-week King’s College Breathlessness Support Service (BSS). Compared with CBIS^13,14^, we found a smaller proportion of WBS participants to derive either ‘no’ (Level 3) benefit (5% WBS versus 8% CBIS) or ‘significant’ (Level 1) benefit (44% WBS versus 56% CBIS), with a greater proportion deriving ‘some’ (Level 2) benefit (41% WBS versus 36% CBIS), as well as some (10%) whose data were difficult to code. Farquhar et al. gave little information on how they applied this scale, raising the possibility that our teams used different criteria. However, leaving aside the distinction between ‘significant’ and ‘some’ benefit, the proportions reporting benefit of either level from CBIS and WBS are similar, and also approximate to the 84% (95% confidence interval: 69–98%) of survey respondents with mixed malignant and non-malignant conditions who reported benefit from the BSS^18^. Taken together, these studies suggest that 80–90% of patients attending breathlessness services may derive benefit of some kind. At the same time, however, this may leave 10–20% of patients who perceive limited benefit but complete the whole programme, potentially wasting resources. It may be possible to improve efficiency through better screening at enrolment and reassessment part-way through to identify patients who are not responding. Our findings broadly agree with quantitative analyses suggesting that patients may be more likely to benefit where they demonstrate needs at enrolment for improving their confidence in managing breathlessness mastery and reducing distress^19^. However, the range of variables involved may make it difficult for formal assessments to improve on a clinical interview of the kind undertaken by the WBS team currently. Also, from an evaluation perspective, the resulting incremental increases in the proportion of patients who benefit are likely to be too small to be reliably detected in RCTs.

Results from qualitative studies to date also raise the question of how future trials of breathlessness services should measure benefits, given how wide-ranging and difficult to quantify these are even when informed by in-depth data. To date, standardised patient-reported outcomes (PROs) have been the most common endpoints in breathlessness service trials^15^. However, these are necessarily limited in their capacity to assess benefit across diverse domains without over-burdening patients with multiple surveys. PROs also typically fail to account for variations in the relative value accorded different domains by different patients. While it may be tempting to use a measure of overall quality of life to address these issues, such measures typically have limited responsiveness to disease-specific interventions^20^. To inform future outcome measurement, trialists are encouraged to include further qualitative sub-studies to provide an ever more nuanced understanding of the breadth and depth of benefits^21^.

Patient feedback on WBS, CBIS and BSS has consistently emphasised the importance, not only of educational content, but its delivery in an individualised way by a caring team^13,14,18^. The current study suggests that one-to-one attention of this kind is not only necessary to understand patient needs and tailor advice, but may also be therapeutic in its own right. Our findings are consistent with previous research showing that patients with COPD may sometimes blame themselves for having caused their disease by smoking and feel ‘unworthy’ of care, with negative impacts on self-management and help-seeking^22,23^. Our results suggest that, for some patients, individual attention from a healthcare team may increase self-esteem, confidence and motivation to engage in self-management as a requisite pathway for improving breathlessness-related outcomes. Moreover, this therapeutic effect appears to depend on patients perceiving that health professionals are genuine, in turn relying on the quality of interaction and commitment of time. Our results suggest that one-to-one person-centred attention from a doctor may have unique capacity in this regard because of experience-based expectations among many patients that medical consultations tend to be brief and biomedical rather than personal in focus. This contribution needs to be considered when interpreting previous research showing evidence of effect from a single-session breathlessness service with no medical involvement^11^.

On a related point, our research highlights how different patients may have varying preferences and needs for educational content and the way this is delivered. Where preferences relate to choice of non-pharmacological strategy or mode of administration, breathlessness services should try to accommodate these through tailoring, especially where delivering unwanted content may result in wasted resources. However, where such preferences indicate a lack of understanding or psychological problems that pose a barrier to effective self-management, they may themselves become a focus for intervention. In particular, our results accord with research conducted across health conditions which suggests that patients may vary in the degree to which they engage as active partners in healthcare, from those who to choose to be as autonomous as possible to those who defer expertise and decision-making to health professionals^24^. The latter pose a special challenge for breathlessness services whose *raison d’être* is to equip patients with the knowledge, confidence and motivation to become consummate in self-management. Much attention has been paid in the literature to promoting a transition for patients who are initially less willing to take an active role in their healthcare, variously described in terms of self-efficacy^25^, activation^26^ and empowerment^27^. Strategies aimed at boosting these requisites have been collectively subsumed under the umbrella term of ‘coaching’ and have been found effective for improving not only self-management but also reducing hospital admissions and even improving health-related quality of life^28^. However, few intervention studies have considered between-patient variability in the need for coaching. Given that coaching is typically the most resource-intensive element of breathlessness self-management interventions, further research is needed to compare approaches that administer education with versus without coaching based on assessment of patient need at enrolment. A more targeted approach may have potential to improve cost-effectiveness for patients who are already self-efficacious (and may therefore benefit just as much from self-management interventions that focus on knowledge transfer and are administered in a low-resource mode (e.g. electronically^29^)), while freeing up resources for patients who need more intensive coaching. Incorporating measures of self-esteem, confidence and motivation into future trials might also help to quantify the relative proportions of patients attending breathlessness services who have these varying needs.

The current study aimed to minimise selection bias by interviewing consecutive WBS patients and continuing to sample until no new information emerged. From an ‘information power’ perspective, our aim was fairly narrow (i.e. focused on WBS rather than experience of breathlessness more generally), our sample specificity dense (i.e. people with COPD and experience of attending WBS), and our analysis more deductive than inductive—all suggesting that a relatively small sample size should be adequate^30^. At the same time, the quality of dialogue was variable given participants’ poor health, and our analysis was strongly cross-case rather than case focused, making it possible that further sampling might have included patients presenting divergent views. Some participants also demonstrated poor memory and mild confusion, limiting confidence in the degree to which they were able to attribute benefits to WBS vis-à-vis pulmonary rehabilitation and other care received. While patients reported valuing the non-judgemental approach taken by WBS to smoking, this may be more reflective of previous poor experiences than be a benefit of WBS itself.

A further potential limitation concerns the risks of social desirability bias and confirmation bias. We attempted to reduce these risks respectively by having an ‘outsider’ with no involvement in delivering the service interview participants and lead the analysis. Questions about potential benefits gave participants the explicit option of answering negatively as well as positively (e.g. ‘did the fan help you or not?’), and participants were only rated as reporting significant global benefit if they could provide concrete examples of how their life had improved rather than stating overall benefit in a generalised way.

In conclusion, this study builds on previous research to suggest that patients with COPD can derive a wide range of benefits from a breathlessness service, over and above pulmonary rehabilitation, that may be difficult to capture comprehensively using a reasonable range of quantitative measures assessing breathlessness severity and/or impact. Participants reported improvements on the affective, impact and (less commonly) sensory-perceptual dimensions of breathlessness. For some participants, these benefits appeared to stem from increases in self-esteem, confidence and motivation, which sometimes extended to other aspects of life and resulted in improved wellbeing more generally. Benefits to self-esteem, confidence and motivation were explicitly attributed by participants to WBS’s long duration, home visits and one-to-one attention from a multidisciplinary team, including a doctor. Further research is needed to test whether including/excluding these more resource-intensive elements based on patient self-esteem, confidence and motivation assessed at enrolment can improve the cost-effectiveness of breathlessness services for people with COPD.

## Methods

The subjective, multi-dimensional nature of chronic breathlessness required a qualitative approach to enquiry with a phenomenological orientation. Reporting of the study has been guided by the Consolidated Criteria for Reporting Qualitative Research^31^. Ethics approval was provided by the Human Research Ethics Committee at Westmead Hospital, Sydney, Australia. All participants gave written informed consent to participate.

### Sample

We interviewed consecutive patients attending WBS between June 2017 and September 2019.

Patients were referred to WBS by Western Sydney respiratory specialists, general practitioners, nurses and allied health personnel. While previous attendance at pulmonary rehabilitation was encouraged, patients who were unable to attend for various reasons were still eligible for enrolment.

Eligible patients were consenting adults (i.e. ≥18 years) who had moderate to very severe COPD (FEV_1_ ≤ 60% predicted; FEV_1_/FVC ratio ≤70%) and severe breathlessness (Modified Medical Research Council Dyspnoea Scale^32^ score ≥2), and were assessed as willing and able to actively participate in their own care by WBS team.

### Data collection

Semi-structured interviews were conducted by telephone upon completion of the service and 6 months later. This article focuses on data from initial interviews only. Interviews focused on perceived benefits of the service, as well as aspects of the service that participants found useful or thought could be improved. Questions focused especially on participant perceptions regarding the service’s duration/frequency, home visits and multidisciplinary delivery, and what (if any) value it added to pulmonary rehabilitation. See Box 1 for a topic guide. Interviews were conducted by a male social scientist with experience in qualitative research (TL), who was employed as a university academic and had no previous or continuing relationships with participants. We explained to participants that our research was aimed at improving WBS for future patients. Interviews were audio-recorded but no field notes were made. Data were transcribed and managed using NVivo version 11 software (QSR International 2015).

Box 1. Interview topic guide

1. What (if anything) did you find helpful about the clinic?*
2. What (if anything) did the clinic offer that was not helpful to you?
3. Is there anything you would change about the breathlessness clinic to make it more helpful?
4. What advice would you give other people with COPD and breathlessness about whether to enrol in the clinic and how to get the most out of it?
5. Do you think the breathlessness clinic would be useful for everyone with breathlessness or only for some people, and why?
6. For people who have completed pulmonary rehabilitation: What (if anything) did you gain from breathlessness clinic over and above your experience at pulmonary rehabilitation?
7. How do you feel now that your involvement in the clinic has finished?

*Follow-up prompts to explore any perceived benefit in more depth:

How (if at all) do you think the clinic helped you:

1. reduce the severity of your breathlessness?
2. reduce your breathlessness unpleasantness?
3. feel more in control of your breathlessness?
4. with your emotional wellbeing and mood?
5. with your ability to get around the house and out and about?
6. with your everyday living?
7. with any other symptoms or problems apart from your breathlessness?

### Analysis

We followed methods described by Farquhar et al.^13,14^ for making global judgements on the degree to which each participant reported benefit from the service. Two researchers (TL and MR) independently applied Farquhar et al.’s 3-point scale of benefit: Level 1—significant impact (clearly stated WBS made a difference); Level 2—some impact (no major change recognised, but valued specific aspects of WBS); Level 3—no impact (WBS made no difference at all). Disagreements were resolved by discussion. Where consensus could not be reached easily, a conservative approach was taken whereby participants were rated as receiving the lower of level of benefit. Where data were considered insufficient or too internally inconsistent for either researcher to make a global judgement, participants were excluded from this part of the analysis.

Thematic analysis used an integrative method designed for informing the development of health service interventions^33^. This method uses both inductive and deductive approaches to build on previous research while remaining open to new insights. The initial coding structure was defined by themes identified in Farquhar et al.’s^13,14^ CBIS qualitative sub-study^13,14^. Benefits to breathlessness were coded according to the dimensions of sensory-perceptual, affective and impact^2^. It was decided that coding of WBS interviews would benefit from both ‘insider’ and ‘outsider’ perspectives to reduce confirmation bias whilst also ensuring that interpretations were informed by a deep understanding of the WBS structure. Analyses were therefore conducted independently by two researchers who then met to discuss any disagreements: TL and a female WBS nurse (MR or VS). Emergent themes were tested for authenticity in subsequent interviews. As interviews yielded less and less information to further develop themes, line-by-line coding was considered unnecessary and only content identified as registering a new theme by the interviewer was subjected to coding by independent researchers. We followed Malterud et al. in defining our sample size according to ‘information power’—a concept similar to saturation but less associated with a specific methodology^30^. In the current study, we determined information power to have been reached when no information warranting addition of new codes was gleaned over five consecutive interviews.

### Reporting summary

Further information on research design is available in the Nature Research Reporting Summary linked to this article.

## Supplementary information

Supplementary Information

Reporting Summary

## Data Availability

In order to protect the identity of participants, interview transcripts cannot be made available.

## Code availability

The complete NVivo 11 coding structure is available on request.
