# Supplementary material for: Patient perspectives on how to optimise benefits from a breathlessness service for people with COPD
Source: NPJ Prim Care Respir Med. 2020 Apr 8;30:16. doi: 10.1038/s41533-020-0172-4 (PMC7142111; doi:10.1038/s41533-020-0172-4)
Supplement: Supplementary file 1 — Supplementary Information [file 41533_2020_172_MOESM1_ESM.pdf]

Supplementary Table 1. The Westmead Breathlessness Service program

Time	Type of contact	Professional	Action
Week 0	Clinic visit, assessment only	Respiratory Physician and Specialist Respiratory Nurse*	Medical review and comorbidity optimisation (including medication assessment)<br>Assessment of breathlessness severity, unpleasantness and ‘confidence’ in managing breathlessness<br>Education regarding the myths of breathlessness<br>Review of strategies already used<br>Introduction of new non-pharmacological strategies<br>Provided with ‘show bag’ containing hand held fan, Breathlessness ‘Action Plans’, written information on non-pharmacological interventions, relaxing and breathing CD and Breathlessness DVD<br>Letter from Respiratory Physician, addressed to patient and copied to involved health care professionals, summarising suggested interventions for management of breathlessness.
Week 1	Phone call	Specialist Respiratory Nurse	Review of clinic assessment and plan

Time	Type of contact	Professional	Action
Week 2	Home visit (carers encouraged to be present)	Occupational Therapist (if required)	Assessment of ADLs and iADLs, home modification referrals, education regarding energy conservation and goal setting**
Week 3	Home visit (carers encouraged to be present)	Physiotherapist (if required)	Breathing techniques, sputum clearance and home exercise program**
Week 4	Home visit (carers encouraged to be present)	Dietitian (if required)	Nutritional assessment and advice**
Week 5	Phone call or home visit	Specialist Respiratory Nurse (if required)	Review of progress (follow up on plan from clinic and goal setting)

Time	Type of contact	Professional	Action
Week 6	Phone call or home visit	Occupational Therapist (if required)	Review of interventions**
Week 7	Phone call or home visit	Physiotherapist (if required)	Review of interventions**
Week 8	Phone call or home visit	Dietitian (if required)	Review of interventions**
Week 9	Clinic visit, assessment only	Respiratory Physician and Specialist Respiratory Nurse*	Medical review including comorbidity optimisation<br>Assessment of breathlessness severity, unpleasantness and 'confidence' in managing breathlessness<br>Review of strategies used<br>Letter from Respiratory Physician, addressed to patient and copied to involved health care professionals, summarising multidisciplinary team interventions and suggestions for ongoing care. Includes table summarising pre/post clinic quantitative outcomes (e.g. chronic

Time	Type of contact	Professional	Action
			respiratory questionnaire subscale results, spirometry results) with explanation of each measure.

*As this clinic is held at a teaching hospital, other health professionals sit in for educational purposes; **Interventions based on individual patient assessment and needs

Supplementary Table 2. Illustrative quotes from participants with moderate to very severe chronic obstructive pulmonary disease (COPD) who had completed an 8 week program with the Westmead Breathlessness Service (WBS).

Results section	Summary	Illustrative quotes
Perceived 'significant' benefit from WBS	Most (n=13/18) participants who were classified as deriving significant benefit (Level 1) cited improvements in activities of everyday living (ADLs) (e.g. housework, self-care, shopping), either in terms of being able to do new activities or perform tasks for a longer duration or with enhanced capability.<br>For a third of participants with significant benefit this flowed on from a perceived reduction in the severity of their breathlessness.	*Now I can clean a room, I clean one room every day. I don't do the floors and that, my husband does all the vacuuming and that, but at least I can cook now every night, and I can dust and I can put the washing on. (P36, 67 year-old woman)*<br><br>*… probably just go a little bit further than you've ever done before, and you just keep increasing things (P39, 73 year-old man)*<br>*P34 (75 year-old man): I'm a mile better than what I used to be.*<br>*INTERVIEWER: So it has actually has made your breathlessness a bit better has it?*<br>*P34: Better than what it used to be.*<br>*INTERVIEWER: That's great. So you don't get as breathless as you used to?*<br>*P34: Just the odd day when it gets too cold.*

Results section	Summary	Illustrative quotes
	Reduction in the impact and affective domains of breathlessness described in terms of greater exercise tolerance, faster recovery, less fear, and increased confidence and ability to cope.	*Yeah, it's being more confident, it's...I found, with the exercising, I don't hesitate to (where, before, I used to wait and wait and wait before I'd) go to the toilet. (P27, 58 year-old woman)*
	Improvements in emotional wellbeing arising from a greater understanding and acceptance of COPD, as well as a sense of being cared for and feeling less alone.	*Generally [I feel] very much more positive. I still have me days … me boom and crash days, but on crash days, it's more, yeah, [I] accept it. I'm not going to do things today - I'm just going to relax. (P4, 67 year-old man)*<br>*Probably one of the most important things is that … [for] anyone who's home alone, is the dreadful feeling of isolation - that if anything happened, you know, no one would've noticed. But that sort of feeling has been ameliorated to a large degree because of the clinic and the various people there. (P28, 68 year-old man)*
	Other reasons that participants were classified as deriving significant impact included reports that they had successfully avoided unnecessary	*It's keeping me out of hospital … I know when it's coming, I feel it coming on and I know that I have to pull myself out of it before it gets too bad. (P18, 73 year-old man)*

Results section	Summary	Illustrative quotes
	ED presentations or hospitalisations as a result of strategies they had learned through the WBS	
Perceived 'some' impact from WBS	Increased comfort in, rather than capacity for, ADLs.	*INTERVIEWER: Has that information enabled you to more than you were doing before do you think, or not?*<br>*P4 (67 year-old man): Not really in the case of doing more, but more the case that I was comfortable in doing it.*
'No' impact	The four participants rated as perceiving no (Level 3, n=3) or inconsistent (difficult to code, n=1) benefit, nonetheless praised the caring attitudes of WBS team members and struggled to identify ways that the service could be improved.	*INTERVIEWER: So it doesn't sound as though there was anything much useful. Was there something else they could have done that would have helped you more, do you think?*<br>*P18 (73 year-old man): No, I don't think so.*
Extension of themes from the CBIS evaluation[1,2]	Breathing techniques and the hand-held fan were perceived to provide psychological	*I feel a lot more confident, I'm not having panic attacks, I'm controlling it better … I stop, sit down, just breath. In and out, in and out. Concentrate,*

Results section	Summary	Illustrative quotes
	benefits as well as positively influencing breathlessness.	*you know? … And you get the fan out, and I settle down and then I'm okay. I get up, and off I go again, and I'm fine. (P29, 68 year-old woman)*
	The indirect nature of benefit and physical challenge associated with exercise made adherence difficult for many participants, with goal setting and motivation from the WBS team members often emphasised as important in enabling this.	*Then I was hospitalised, and I just sat for two years. Then, getting me up and giving me goals - my steps for the day, and my exercises - I kept it going, and I'm feeling a lot better. (P27, 58 year-old woman)*
	Substantial emphasis placed on the supportive 'can do' attitude of the WBS team.	*They've got an amazing service there. They're all very, very dedicated people to their style of medicine, and they do help a lot. They support you immensely. They're always pleased to see you, and they're just a pleasure to be around, actually. They're quality people. (P20, 61 year-old man)*
Patient perceptions of WBS versus pulmonary rehabilitation	WBS program was perceived to cover a greater variety of therapies in more depth, be more personalised, involve caregivers more, and go	*The Breathless Clinic is a much more personal experience (than pulmonary rehabilitation), because they come to your home and they're talking to you*

Results section	Summary	Illustrative quotes
	beyond the physical aspects of COPD to more holistically address wellbeing and consider comorbidities.	*direct, your wife; your partner becomes more involved and understands, and that helps both parties. (P1, 75 year-old man)*
Patient perceptions regarding home visits	Around half (n=20) of participants perceived home visits to be an important feature of the service both for improving access and enabling team members to assess and remediate ADLs in situ. Two participants asserted that they could not have attended the service if it had been run exclusively at the hospital due to logistic problems with transport, limitations in mobility/physical functioning, or anxiety related to travel or hospitals.	*If she [nurse] didn't come out, I wouldn't be able to go in - it's as simple as that. Even just the thought of it [going into hospital], and my butterflies start to increase, and then I get the dizziness. (P24, 72 year-old man)*

Results section	Summary	Illustrative quotes
	However, there were two exceptions, who reported feeling overwhelmed by the number and frequency of home visits.	*[At home visit time] it was just, "oh, who've we got today? How many today?" I just felt it was too confronting. It was too many. (P8, 70 year-old woman)*
Patient perceptions of WBS's individual focus	Participants often cited the individualised nature of the WBS as beneficial for enabling the WBS team to develop a deep understanding of their individual needs required to tailor advice, and to build personal relationships with them that communicated care and support and encouraged disclosure. Experience of being cared for and supported as an individual was among the most commonly emphasised feature of the service and perceived by many to be a therapeutic element in its own right.	*They're very caring people. And they do understand, they don't make you feel bad. (P19, 54 year-old woman)*<br><br>*[WBS] gave me a lot because they encourage you to do better for yourself, and they really do care. (P20, 61 year-old man)*

Results section	Summary	Illustrative quotes
	The characteristics of care and support that were most commonly accentuated included genuineness, lack of time constraint, understanding about the impact of breathlessness, respect and dignity, and lack of judgement regarding their smoking history. In some cases, this was contrasted with a lesser sense of support they had experienced in other healthcare encounters.	*P15 (59 year-old woman): Even though I still smoke, they didn't judge me. They were helping me [to give up], if that makes sense?*<br>*INTERVIEWER: Yes, and is that different to your experience from some other healthcare services?*<br>*P15: Yes. My experience just with seeing other medics and my heart specialist - you can sense them [trails off].*<br><br>*If you feel down, they'll go, "you're just as worthy as [trails off]". (P38, 60 year-old man)*
	While two participants reported benefitting from socialising with other participants in previous group programs, all who were asked considered the benefits of one-to-one time to outweigh this.	*INTERVIEWER: And do you think that [being seen one-to-one] was good, or would you have liked some group things as well?*<br>*P7 (62 year-old man): No, no. It was good to be seen [one-to-one]. I wanted the time … some of it's pretty personal stuff, I guess.*

Results section	Summary	Illustrative quotes
Patient perceptions regarding WBS's program duration	Most who were asked expressed approval regarding the duration of the program	*It [duration] was about right, because you needed that time for you to engage there with people that could actually help. (P24, 72 year-old man)*
	Two participants would have preferred a longer duration	*I would've liked for it to have gone on a little bit longer, but the 8 weeks that I did have were vital, absolutely. (P20, 61 year-old man)*
	Three participants perceived the content to be unnecessarily repetitive and/or include content not relevant to them.	*The only thing I can say is that it's just the repetition of the questions. But everyone has their own little folders, and writing things in, you think "oh gosh, not again", but that's the only thing. (P2, 84 year-old woman)*
		I think it was helpful, and I don't want to deter anybody else that needs it. I just think for me in particular, I think I'm just too ... Okay, I'll be honest, and I don't mean this rudely, but I think I outdid what they wanted me to do. You know what I mean? Like, I was way ahead of what they were all planning me to do. (P12, 69 year-old woman)

Results section	Summary	Illustrative quotes
	However, others perceived repetition to be useful for consolidation and reinforcement.	*I think each and every one of them would come back to something about the pursed lips … constantly from everyone. It was emphasized more, you know what I mean? (P8, 70 year-old woman)*
	Many participants stressed the importance of knowing that the WBS team was available by telephone after the sessions were completed to give them a sense of continuing support and safety in the event of future problems.	*I've got their numbers if I need to call them for anything at any time. It hasn't just stopped. They said to me, "it keeps going - if you need us, we're here". (P27, 58 year-old woman)*
Patient perceptions of WBS multidisciplinary input	Participants varied in the degree to which they distinguished the roles and contributions of different disciplines within the WBS team.	*INTERVIEWER: Did you like that? That you had a doctor, a nurse, you had different types of health professional involved.*<br>*P15 (59 year-old woman): Yes, I did because I didn't realize that COPD involves nutrition, walking, exercise. Does that make sense?*

Results section	Summary	Illustrative quotes
		I don't know the dietician. The occupational therapist was the one that told me how to breathe properly and do the exercises? (P17, 81 year-old woman)
	Some participants perceived that team members' different skillsets worked together both by focusing on different aspects of care and reinforcing key messages.	*You get someone like the dietitian ... she knew exactly what she was talking about. She wasn't taking information from someone else and then passing it on … It's the same with the physio; she was a qualified physio, not just a nurse trying to act as a physio. (P14, 76 year-old man)*
	Even where participants seemed less clear on the role of each discipline, collective attention from a number of health professionals increased their sense of being cared for and motivated them to make more effort to self-manage. In this way, participants drew an explicit causal pathway from the team's caring and supportive approach through to their	*You realise that all those people are out there and all they want to do is help you … your ego, they boost you, your spirit. It gives you a bigger incentive to get on with it. So, yes - it certainly helped so much. (P6, 74 year-old man)*

Results section	Summary	Illustrative quotes
	capacity to self-manage and resulting impacts on the dimensions of breathlessness.	
	Changes in participant self-esteem, confidence and motivation seemed not to be limited to breathlessness self-management but impacted their approach to life more generally.	*P3 (69 year-old man): Percentage wise, even though I've been through two rehabs down at [name of hospital], percentage wise, I'd say that I was only doing about 25%, if that. And I'm now trying to push 95% of all the things … And I'm setting myself goals that I want to achieve, and I'm trying to be as determined as I bloody can … I personally think that - and I didn't even ask 'em about this - but being determined, determination, is going to be 75% of the battle.*<br>*INTERVIEWER: So it sounds like they've gone beyond the breathlessness a bit to help you with some other things as well?*<br>*P3: They've gone all of life, I'd say.*
	While all the disciplines were singled out for praise by at least some participants, participants seemed especially impressed with	*I must say, I've never met a doctor that is so supportive. And she's so caring, and she really is so supportive that when she looked at all the things I was doing, she said it was all me, and she was very proud of what I've*

Results section	Summary	Illustrative quotes
	the time commitment and level of care shown by the doctor.	*been achieving. I can't explain, I've just never met a doctor like her. She doesn't put you down, she doesn't rush you along like some doctors do. You go in, and then they [other doctors] want you out in five or ten minutes. (P36, 67 year-old woman)*
	Participants also highlighted the unique role the doctor played in prescribing medications (including low-dose opioids), ordering and interpreting tests, and using medical authority to enable access to healthcare for comorbid conditions and disability benefits.	*You know, and having their [the doctor's] expertise, saying "no, we['ve] got to get rid of that [medication]", and go from there … the doctor has the expertise to do all of that, and give you a script for whatever it is you … like, I was experiencing a very, very, very dry mouth, so we were able to change the puffers to accommodate that. (P33, 74 year-old man)*
	Although not explicit, there was a sense that the doctor's opinion carried special weight, especially on sensitive matters such as the need for referral to a psychologist or advance care planning.	*[Advance care planning] has been explained to me, what can happen … And I'm currently talking to the local GP, family and friends about it, this type of thing. This is all due to Doctor [name] and her crew. (P4, 67 year-old man)*

Results section	Summary	Illustrative quotes
	There were also three negative cases who seemed not to perceive substantial differences in the roles of WBS team members. For the participant with no global benefit, this extended even to the doctor's role.	*I've got to be honest … she [the doctor] made no difference. Whether it was the doctor or just nurses or physio, it was just like a team of people to me, trying to help you. (P30, 68 year-old woman)*
Patient suggestions for improving WBS	When asked how to improve WBS, more than half (n=25) responded by saying this would be difficult to do because quality was already so high.	*I don't know how they could make it better because they're all so caring and supportive. (P36, 67 year-old woman)*
	Everyone who volunteered improvements (n=16) cited problems with travelling to/from the hospital due to a lack of means of transport or difficulty parking. While home visits were perceived to partially offset these problems,	*Going to the hospital is a real drama isn't it? First starts with that big dirty word starting with 'P' - parking. (P26, 75 year-old man)*<br><br>*I can drive, but unless I can get a disability spot I have to park too far away, and I can't walk that far, so it's a catch-22. I have to rely on my*

Results section	Summary	Illustrative quotes
	hospital visits were still required for initial assessment and one follow-up.	*friend, who's not always available, and she's not my taxi service. (P25, 71 year-old woman)*
	At least one participant was unaware of the free patient transport service available to/from the hospital.	*If you get transport, how much [do] they charge or would they wait for you? (P16, 80 year-old woman)*

ADLs = activities of daily living; CBIS = Cambridge Breathlessness Intervention Service; WBS = Westmead Breathlessness Service

References

1 Farquhar, M. C. *et al.* Is a specialist breathlessness service more effective and cost-effective for patients with advanced cancer and their carers than standard care? Findings of a mixed-method randomised controlled trial. *BMC Med* **12**, 194, doi:10.1186/s12916-014-0194-2 (2014).

2 Farquhar, M. C. *et al.* The clinical and cost effectiveness of a Breathlessness Intervention Service for patients with advanced non-malignant disease and their informal carers: mixed findings of a mixed method randomised controlled trial. *Trials* **17**, 185, doi:10.1186/s13063-016-1304-6 (2016).
